# Internalization of Metal–Organic Framework Nanoparticles in Human Vascular Cells: Implications for Cardiovascular Disease Therapy

**DOI:** 10.3390/nano10061028

**Published:** 2020-05-27

**Authors:** Dana E. Al-Ansari, Nura A. Mohamed, Isra Marei, Atef Zekri, Yu Kameno, Robert P. Davies, Paul D. Lickiss, Md Mizanur Rahman, Haissam Abou-Saleh

**Affiliations:** 1 Department of Biological and Environmental Sciences, Qatar University, Doha 2713, Qatar; da1404794@qu.edu.qa (D.E.A.-A.); nura.adam@qu.edu.qa (N.A.M.); mrahman@qu.edu.qa (M.M.R.); 2 Department of Pharmacology, Weill Cornell Medicine-Qatar, Doha 24811, Qatar; isra.marei11@imperial.ac.uk; 3 Cardiothoracic Pharmacology, National Heart and Lung Institute, Imperial College London, London SW7 2AZ, UK; 4 Qatar Energy and Environment Research Institute, Hamad Bin Khalifa University, Qatar Foundation, Doha 34110, Qatar; azekri@hbku.edu.qa; 5 Department of Chemistry, Imperial College, London, White City Campus, 80 Wood Lane, London W12 0BZ, UK; y.kameno16@imperial.ac.uk (Y.K.); r.davies@imperial.ac.uk (R.P.D.); p.lickiss@imperial.ac.uk (P.D.L.); 6 Biomedical Research Center, QU Health, Qatar University, Doha 2713, Qatar

**Keywords:** nanomedicine, cardiovascular diseases, metal–organic framework nanoparticles, MIL-89, endothelial cells, smooth muscle cells

## Abstract

Cardiovascular diseases (CVDs) are the leading cause of morbidity and mortality worldwide. Alteration of endothelial cells and the underlying vasculature plays a central role in the pathogenesis of various CVDs. The application of nanoscale materials such as nanoparticles in biomedicine has opened new horizons in the treatment of CVDs. We have previously shown that the iron metal–organic framework nanoparticle, Materials Institut Lavoisier-89 (nanoMIL-89) represents a viable vehicle for future drug delivery of pulmonary arterial hypertension. In this study, we have assessed the cellular uptake of nanoMIL-89 in pulmonary artery endothelial and smooth muscle cells using microscopy imaging techniques. We also tested the cellular responses to nanoMIL-89 using molecular and cellular assays. Microscopic images showed cellular internalization of nanoMIL-89, packaging into endocytic vesicles, and passing to daughter cells during mitosis. Moreover, nanoMIL-89 showed anti-inflammatory activity without any significant cytotoxicity. Our results indicate that nanoMIL-89 formulation may offer promising therapeutic opportunities and set forth a new prototype for drug delivery not only in CVDs, but also for other diseases yet incurable, such as diabetes and cancer.

## 1. Introduction

Cardiovascular diseases (CVDs) are the leading cause of death and a major cause of disability worldwide [1]. Even though many types of CVDs are well diagnosed and treated, other forms are still hard to detect and manage, while others are incurable. In fact, some CVDs have many challenges to overcome, such as maintaining the pharmacoefficacy and the stability of the therapeutic agent in the body, as well as avoiding the systemic side effects caused by the unnecessary accumulation in different organs and tissue [2]. This reinforces the need to explore new therapeutic strategies to overcome the limitations of conventional therapeutics. Nanomedicine is defined as the employment of nanoparticles (NPs, i.e., particles with a diameter between 1 and 150 nm) often as drug delivery vehicles or for imaging disease pathology and progression [3,4]. In the last four decades, the amount of nanomedicine research has considerably increased [5]. The cancer field has benefited the most from nanomedicine, with over 7000 hits for ‘oncology’ and ‘nanomedicine’ on PubMed [6]. However, other medical fields should benefit from the advances made in the cancer nanomedicine field, and use the features of cancerous tissues that can be seen in other diseases, such as CVDs, to their advantage. Some examples of these cancerous features are damaged tissues and remodeled vessels, which lead to the formation of heavily vascularized tissues with more blood flow, leading to the accumulation of NPs in sites of injury, a mechanism referred to as passive targeting delivery [7,8]. Furthermore, the significant structural abnormalities in the area surrounding the cancerous tissue, referred to as the tumor microenvironment, enhance the chances for more passive targeting delivery to take place. Moreover, the enhanced permeability and retention properties of the cancerous tissues will allow for more NPs to be internalized by the cells [9]. Many forms of CVDs share these features with cancer, which suggests that the advancements made in cancer nanomedicine may be applied in the cardiovascular field [10]. Hence, the emerging field of cardiovascular nanomedicine (CVN) is now showing promising lab-scale results [11]. Examples of CVN research areas include hypertension [10], atherosclerosis [12], thrombosis [13], inflammation [14,15], hyperlipidemia, stroke [10], myocardial infarction [16] and pulmonary arterial hypertension (PAH) [17,18]. In particular, PAH is gaining more attention as it is an aggressive disease with poor prognosis, no available cure, and low survival rates. The etiology of PAH is complex and involves intricate and multifactorial processes. A hallmark finding is the dysfunction and proliferation of pulmonary artery endothelial (PAECs) and smooth muscle (PASMCs) cells, leading to vascular remodeling and loss of small pulmonary arteries [19,20]. Taking into account that early detection and targeted therapy are the most difficult challenges in PAH, it is clear that developing nanoparticles that can be tracked (in situ) using non-invasive procedures will be highly beneficial [21].

Of the available NPs, MIL-89 (nanoMIL-89), a family of highly porous, iron-based metal–organic frameworks (MOFs), is found to have several advantages over other nanoparticles, such as (i) low density, (ii) biocompatibility and (iii) high drug loading capacity [11,22,23]. We have previously developed an enhanced formulation of nanoMIL-89 and characterized its physio-chemical proprieties and stability. NanoMIL-89 has also shown relative safety and tolerability [15]. However, the cellular internalization and fate have still not been investigated. When introducing a new nanomaterial for biomedical applications, it is first essential to determine its interaction and cellular responses within the cells relevant to the studied disease. Therefore, in this study, we assessed the cellular uptake and cytoplasmic localization of nanoMIL-89 in artery endothelial and smooth muscle cells, the major culprits involved in the pathogenesis of CVDs, including PAH. Moreover, we tested the effects of nanoMIL-89 on cell viability and inflammatory responses, to highlight the safety and efficacy of iron-based MOF nanoparticles as future therapeutic tools in many CVDs.

## 2. Materials and Methods

### 2.1. Preparation and Chemical Analysis of NanoMIL-89

NanoMIL-89 was prepared following the previously published procedure by Mohamed et al. [23], and then characterized using Powder X-ray Diffraction (PXRD; Bruker D2 Phaser, Coventry, UK) and Dynamic Light Scattering (DLS; DelsaNano C by Beckman Coulter, High Wycombe, UK) to confirm that the PXRD pattern and DLS size are consistent with published data.

### 2.2. Cell Lines

Using standard cell culture techniques described previously [23], human pulmonary artery endothelial cells (PAECs; PromoCell, Heidelberg, Germany) and human pulmonary artery smooth muscle cells (PASMCs; ScienCellTM, San Diego, CA, USA) were plated and maintained in culture according to the supplier’s recommendations.

### 2.3. Cell Culture

PAECs and PASMCs were cultured, as described previously [23]. Briefly, PAECs and PASMCs were seeded at a density of 10,000 cells and 100,000 cells per well, respectively. Cells were then incubated overnight (16–18 h) at humidified conditions (37 °C, 95% O_2_, 5% CO_2_). Following that, cells were treated with different concentrations of nanoMIL-89 (0–100 µg/mL) in normal and inflammatory conditions. The inflammatory condition was induced by treating the cells with 1 µg/mL lipopolysaccharide (LPS).

### 2.4. Viability, Cytotoxicity and Cytokine Release Assays

PAECs and PASMCs were treated with different concentrations of nanoMIL-89 in normal and inflammatory conditions and kept in culture for 24 h. The supernatants were then collected and used for measurements of Lactate Dehydrogenase (LDH) (Invitrogen^TM^, Carlsbad, CA, USA) and C-X-C Motif Chemokine Ligand 8 (CXCL8) release (IL8/CXCL8 DuoSet ELISA, R&D System, Minneapolis, MN, USA) following the manufacturer’s instructions. Adherent cells were used to perform a viability test using alamarBlueTM (Invitrogen^TM^, Carlsbad, CA, USA) following the manufacturer’s instructions. 

### 2.5. Light Microscope Imaging and Blind Scoring

To investigate the internalization and intracellular localization of NPs, cells were incubated with different concentrations of NPs (0–100 µg/mL) in normal and inflammatory conditions, and images were taken using an inverted light microscope (EVOS XL, Advanced Microscopy Group, Mill Creek, WA, USA) at 1, 3 and 7 days. Blinded visual scoring of images was performed by 6 experienced researchers that were blinded to the treatment group in order to avoid unintentional bias in scoring. Images were scored from 0 to 5 based on the absence (0) or the presence (1–5) of NPs in the cell cytoplasm. The transfer of internalized NPs from mother to daughter cells was determined by imaging a population of dividing cells at different time points (1, 3 and 7 days).

### 2.6. Confocal Microscope Imaging

PAECs and PASMCs suspensions were pre-stained with 5 µM CellTrackerTM Red CMTPX Dye (Invitrogen^TM^, Carlsbad, CA, USA) as instructed by the manufacturer. Cells were seeded into gelatin-coated coverslips at a density of 6 × 104 cells/well and were allowed to adhere overnight under humidified conditions. Cells were then treated with 3 and 10 µg/mL of nanoMIL-89 for 24 h and fixed with 4% Paraformaldehyde (Thermo Scientific™, Waltham, MA, USA) for 10 min and stained with diamidino-2-phenylindole (DAPI) (Invitrogen^TM^, Carlsbad, CA, USA) and Alexa Fluor™ 488 Phalloidin (Invitrogen^TM^, Carlsbad, CA, USA). Samples were imaged using Carl Zeiss LSM-880 confocal microscope. Phase-contrast images were obtained using Zen black software, and 2D images were acquired using red (Ex: 581 nm/Em: 609 nm), blue (Ex: 358 nm/Em: 461 nm), and green (Ex: 495 nm/Em: 518 nm) filters. 3D focus stacking images (Z-stack) and 3D projections were generated to visualize NPs’ internalization within the cells using Zen blue software. Orthogonal projections (Frontal; xy, Transverse; xz, and Sagittal; yz) were also generated using Zen blue software (V 2.3 lite), (Figure S1). Sliced 3D images were generated using Imaris imaging software (IMARIS 8, Oxford Instruments, Abingdon, USA).

### 2.7. Sample Preparation for Scanning Transmission Electron Microscope and EDS Analysis

PAECs and PASMCs were seeded in 100 mm culture dishes with regular media changes until confluent cell monolayers were formed. PAECs and PASMCs were then treated with 5 and 3 µg/mL of nanoMIL-89, followed by 24 h incubation. Post-treatment, the cells were trypsinized and fixed with equal amounts of 2% glyceraldehyde and 4% paraformaldehyde in PBS and centrifuged, forming a tight pellet. After 2 h of fixation, the initial fixative was replaced with 8% sucrose. Pellets were then post-fixed with 1% OsO4 in 0.1 M PBS for 1 h and then dehydrated gradually with acetone (50%–100%). Pellets were then embedded in Agar 100 epoxy resin (Agar Scientific Ltd., Essex, UK) with the following formulation: 24 g Agar 100 epoxy resin, 16 g dodecenylsuccinic anhydride, 10 g methyl nadic anhydride and 1.5 g benzyldimethylamine. The epoxy resin was then incubated overnight at 60 °C, forming a block with medium hardness. The blocks were then sectioned using Leica EM UC7 ultramicrotome system (Leica Microsystems Wetzlar, Germany) into ultra-thin sections (~<100 nm). The sections were retrieved from water surface and mounted on a 200-mesh copper grid. To improve the contrast and sample stability under electron beam, the sections were coated with carbon before imaging. Sections were then imaged using scanning transmission electron microscope (STEM), and the intracellular signal of iron (Fe) was measured using Bruker energy dispersive spectroscopy (EDS) (Billerica, MA, USA) installed in a Talos TEM system (FEI, Hillsboro, OR, USA).

### 2.8. Statistical Analysis

Data were presented as mean ± SEM for n independent experiments using GraphPad Prism 8 (GraphPad Prism Software, San Diego, California, USA). *p* * < 0.05 was considered statistically significant.

## 3. Results

### 3.1. Chemical Characterization of NanoMIL-89

**Figure 1:**
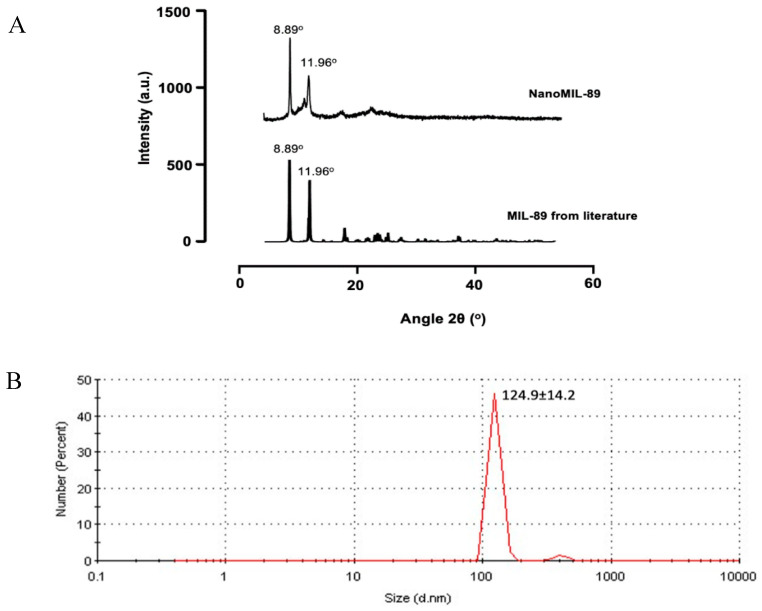
Characterization of nanoMIL-89 using (**A**) Powder X-ray Diffraction (PXRD) comparing the synthesized nanoparticle pattern [25] with the Materials Institut Lavoisier-89 MIL-89 pattern reported in the literature by literature by Horcajada.et.al (2006). (**B**) nanoMIL-89 estimated gyration diameter using Dynamic Light Scattering (DLS) analysis.

The synthesized product was characterized using PXRD analysis and DLS. The PXRD pattern was similar to the previously published pattern by our lab [24] and others [25,26] (Figure 1A). DLS analysis of four consecutive nanoMIL-89 batches in ethanol, as it is the solvent used in the nanoparticle synthesis, showed an estimated gyration diameter of 146.4 ± 17.4 nm, 133.0 ± 14.2 nm, 137.8 ± 13.6 nm, and 124.9 ± 14.2 nm (Figure 1B). These results indicate that the nanoMIL-89 prepared in this study had the expected PXRD pattern and an appropriate nanoparticle size for use in biological studies (i.e., <150 nm).

### 3.2. Effects of NanoMIL-89 on Cell Viability and Toxicity

**Figure 2:**
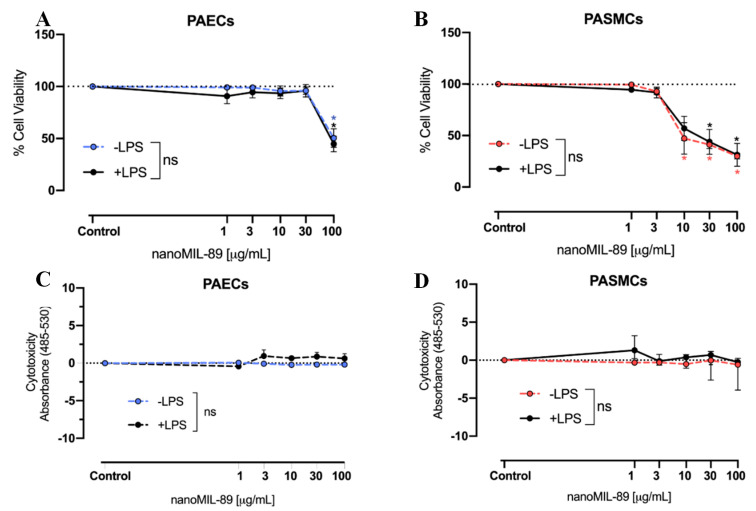
Effect of nanoMIL-89 on cell viability and toxicity. Cells were incubated in (−LPS/+LPS) culture media and treated with nanoMIL-89 (0, 1, 3, 10, 30,100 µg/ml). (**A**,**B**) Effect of nanoMIL-89 on the viability of PAECs and PASMCs. (**C**,**D**) Toxicity effect of nanoMIL-89 on PAECs and PASMCs. Data are mean ± SEM for *n* = 3. Statistical analysis between different groups (−LPS/+LPS) was determined by two-way Analysis Of Variance (ANOVA) followed by Bonferroni posttests (* *p*  <  0.05). Statistical analysis for each condition compared to the relevant controls was determined by one-way ANOVA, followed by Dunnett’s multiple comparison post-test (* *p*  <  0.05).

Figure 2 depicts the effects of nanoMIL-89 on cell viability and toxicity. At low to moderate concentrations (< 30 µg/mL), nanoMIL-89 did not affect the PAECs’ viability, as measured by alamarBlueTM viability reagent under normal and inflammatory conditions induced by LPS 1 µg/mL (Figure 2A). PASMCs, on the other hand, were more sensitive to the effect of nanoMIL-89 as there was a reduction in cell viability at concentrations > 10 µg/mL under normal and inflammatory conditions. The decrease in cell viability was concentration-dependent, with approximately 50% reduction in cell viability observed with a nanoMIL-89 concentration of 100 µg/mL (Figure 2B). To investigate further whether this reduction in cell viability is associated with cytotoxicity, LDH assay was performed. Results showed that there was no significant release of LDH in either PAECs or PASMCs, at the concentrations of nanoMIL-89 tested (Figure 2C,D).

### 3.3. Anti-Inflammatory Effect of NanoMIL-89

**Figure 3:**
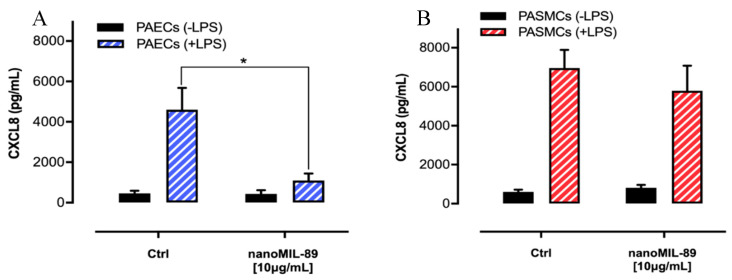
Effect of nanoMIL-89 on the release of C-X-C Motif Chemokine Ligand 8 (CXCL8) from (**A**) PAECs and (**B**) PASMCs. Cells were cultured in (−LPS/+LPS) media and treated with nanoMIL-89 (10 µg/mL). Data are mean ± SEM; *n* = 3. Statistical analysis was carried out by two-way ANOVA followed by Bonferroni Posttests (*p* * < 0.05).

The anti-inflammatory effect of nanoMIL-89 was assessed by measuring the release of the cytokine CXCL8 from cells treated with LPS, the most abundant component within the cell wall of Gram-negative bacteria [27]. As indicated in Figure 3, the basal release of CXCL8 was significantly increased following treatment with 1% LPS in PAECs (459.37 ± 132.42 pg/mL vs. 4604.07 ± 1074.9 pg/mL) and PASMCs (602.2 ± 113.6 pg/mL vs. 6960.9 ± 927.5 pg/mL). When treated with 10 µg/mL of nanoMIL-89, PAECs showed a significant reduction of CXCL8 release (1089 ± 454.5 pg/mL), whereas CXCL8 release from PASMCs was not significantly affected.

### 3.4. Internalization and Subcellular Localization of NanoMIL-89

#### 3.4.1. Light Microscope

**Figure 4:**
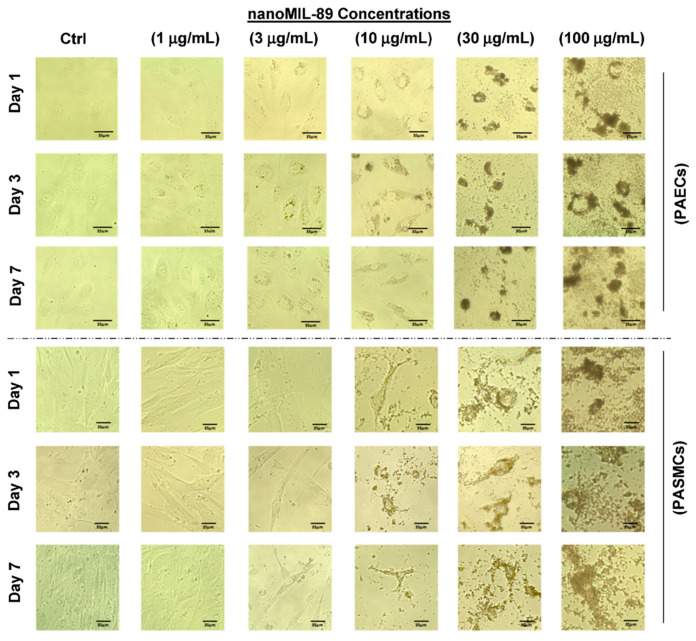
nanoMIL-89 uptake by PAECs and PASMCs. Cells were treated with different nanoMIL-89 concentrations (0, 1, 3, 10, 30 and 100 µg/mL). Images were taken using an inverted light microscope (magnification 20×) at days 1, 3 and 7.

**Figure 5:**
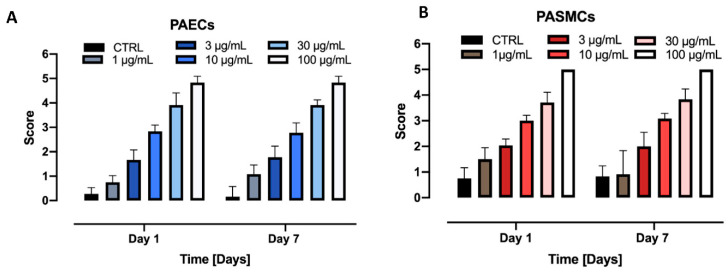
Blind scoring of nanoMIL-89. Cells were treated with different concentrations of nanoMIL-89 (1, 3, 10, 30, 100 µg/mL) and imaged with inverted light microscope. Cellular uptake of nanoMIL-89 by (**A**) PAECs and (**B**) PASMCs was determined by blinded visual scoring (*n* = 6 independent assessments) according to a relative scale from 0 to 5.

**Figure 6:**
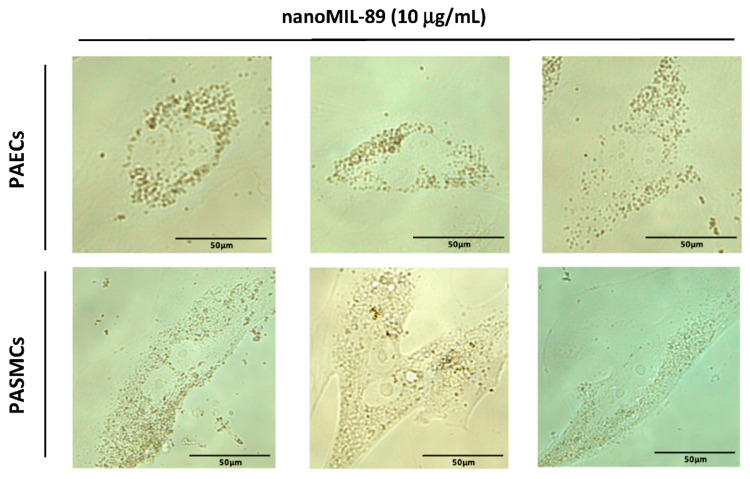
Internalization and fate of nanoMIL-89. Photomicrographs showed the transfer of internalized NPs from mother to daughter cells during mitosis. Images were taken using inverted light microscope (magnification 40×) and cells were imaged during the division stage at different time points.

The uptake of nanoMIL-89 by PAECs and PASMCs was first assessed by inverted light microscopy at different time points (1, 3 and 7 days), under normal and inflammatory conditions induced by LPS (Figure 4 and Figure S1). As shown in Figure 4, nanoMIL-89 uptake increased with time in a concentration-dependent manner in both cell types. The NPs appeared to be localized in the cytoplasm of the cells, with increased nanoparticle numbers as the concentration increased. At low concentrations (≤10 µg/mL), PAECs presented a normal cobblestone endothelial cell morphology and longitudinal alignment. However, at the highest concentrations (≥30 µg/mL), cells exhibited an unhealthy circular appearance and started to detach from the plate due to the increased uptake of nanoMIL-89. Moreover, PASMCs were observed to be more sensitive to nanoMIL-89 as they start deforming from their fusiform spindle shape at ≥10 µg/mL. This is consistent with our viability/cytotoxicity findings showing that high concentrations of nanoMIL-89 resulted in reduced viability of these cells. Moreover, LPS does not seem to affect cell permeability and subsequent nanoMIL-89 uptake. The cellular uptake was further assessed by blinded visual scoring, and samples were scored from 0 to 5 based on the quantitative distribution of NPs in the cell cytoplasm. The results obtained confirm that nanoMIL-89 were taken up by PAECs and PASMCs in a concentration-dependent fashion (Figure 4 and Figure 5) in basal conditions as well as under inflammatory condition (Figures S2 and S3). Interestingly, we found that internalized NPs were further passed on from mother to daughter cells during mitosis (Figure 6).

#### 3.4.2. Confocal Microscope

**Figure 7:**
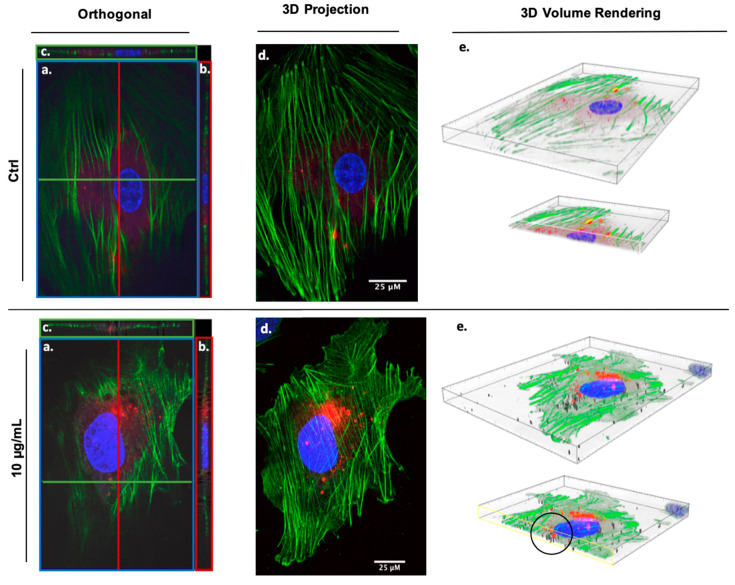
Z-stack images showing nanoMIL-89 uptake in PAECs. Orthogonal and 3D projections of Z-stack images of pre-stained PAECs showing: Untreated (control) PAECs and PAECs treated with 10 µg/mL nanoMIL-89. Projections are: (**a**) Transverse (xz, blue), (**b**) Sagittal (yz, red), (**c**) Frontal (xy, green), (**d**) 3D projection of Z-stack images and (**e**) 3D volume rendering of a single cell with a sliced view showing the cell content. Channels used—Red: CellTrackerTM Red CMTPX Dye, Green: phalloidin, Blue: diamidino-2-phenylindole (DAPI), and phase contrast: nanoMIL-89. Images indicate that nanoMIL-89 are internalized within the cytoplasm of the cells (black circle). Scale bar is 25 µm.

**Figure 8:**
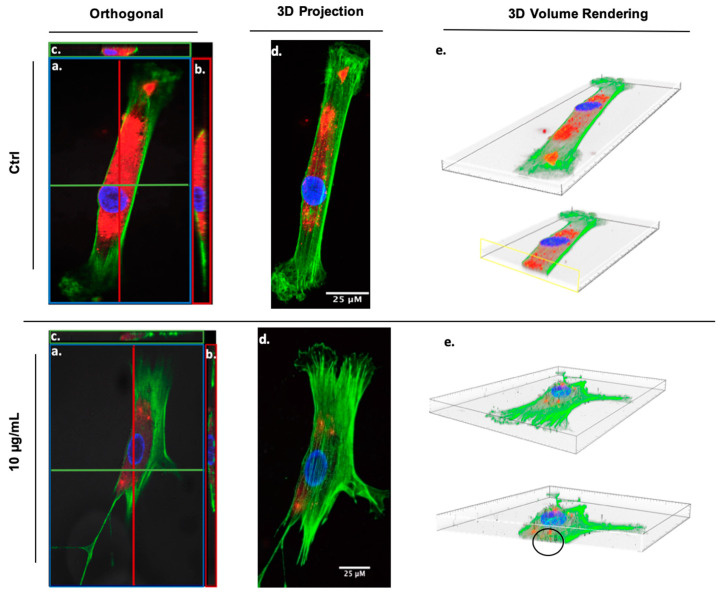
Z-stack images showing nanoMIL-89 uptake in PASMCs. Orthogonal and 3D projections of Z-stack images of pre-stained PASMCs showing: Untreated (control) PASMCs and PASMCs treated with 10µg/mL nanoMIL-89. Projections are: (**a**) Transverse (xz, blue), (**b**) Sagittal (yz, red), (**c**) Frontal (xy, green), (**d**) 3D projection of Z-stake confocal images and (**e**) 3D volume rendering of a single cell with a sliced view showing the cell content. Channels used—Red: CellTrackerTM Red CMTPX Dye, Green: phalloidin, Blue: DAPI, and phase contrast: nanoMIL-89. Images indicate that nanoMIL-89 are internalized within the cytoplasm of the cells (black circle). Scale bar is 25 µm.

The confocal microscope was used to further characterize the internalization and subcellular localization of NPs within the cells. 3D images of cells treated with 10 µg/mL of nanoMIL-89 were obtained (Figure 7 and Figure 8). The images show the intracellular localization of NPs within the cytoplasm of both PAECs and PASMCs.

#### 3.4.3. Scanning Transmission Electron Microscope and EDS Analysis

**Figure 9:**
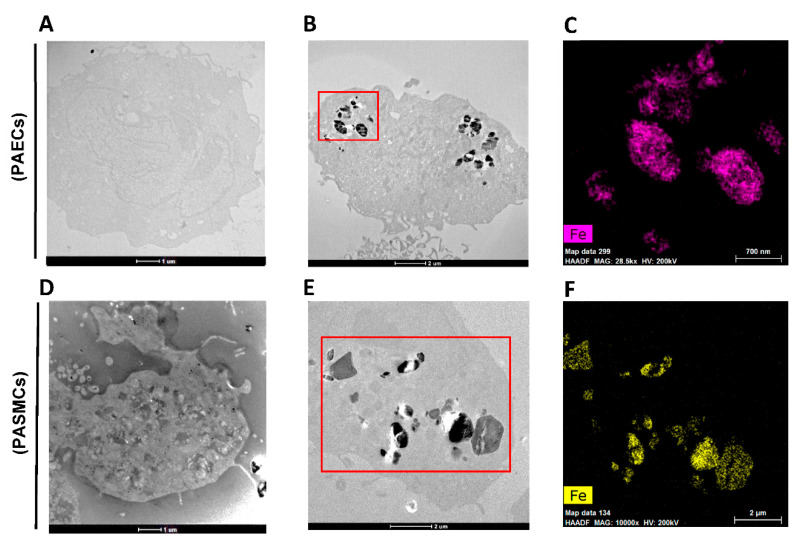
Scanning Transmission Electron Microscope images of PAECs and PASMCs treated with 5µg/mL nanoMIL-89. Cells embedded in Epoxy resin sections (around 100 nm) showing (**A**) an empty PAECs (scale bar = 1 µm), (**B**) nanoMIL-89 incorporated in PAECs (scale bar = 2 µm) and (**C**) energy dispersive spectroscopy (EDS) elemental mapping showing iron distribution inside the nanoMIL-89-treated sections (red square) of (**B**) PAECs (scale bar = 700 nm). (**D**) empty PASMCs (scale bar = 1 µm), (**E**) nanoMIL-89 incorporated in PASMCs (scale bar = 2 µm), and (**F**) EDS elemental mapping showing iron distribution inside the nanoMIL-89-treated sections (red square) of (**E**) PASMCs (scale bar = 2 µm).

**Scheme 1:**
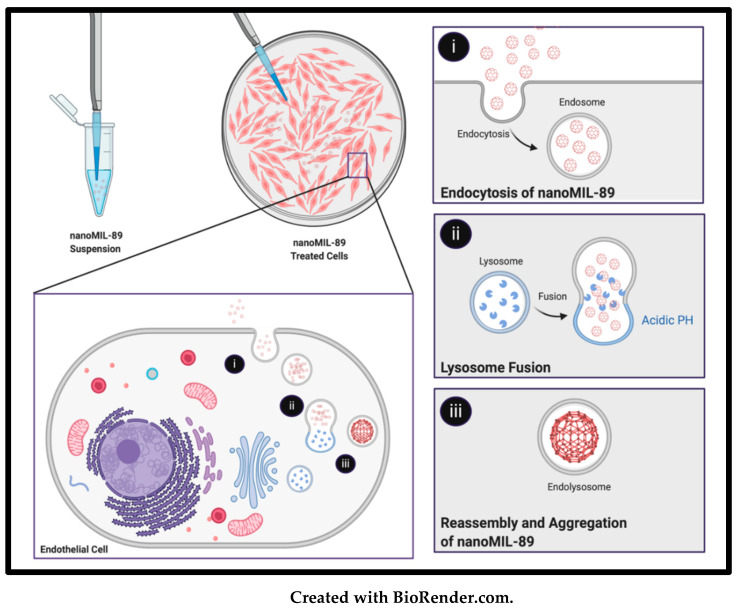
Illustration of the proposed mechanism of nanoMIL-89 cellular uptake and internalization into endocytic vesicles. (**i**) NanoMIL-89 were uptaken by the cells by endocytosis, packaged into an endosome, and (**ii**) fused with a lysosome, forming (**iii**) an endolysosome vesicle. The reassembly and aggregation of nanoMIL-89 is triggered by the acidic environment within the interior membrane of the lysosome.

**Figure 10:**
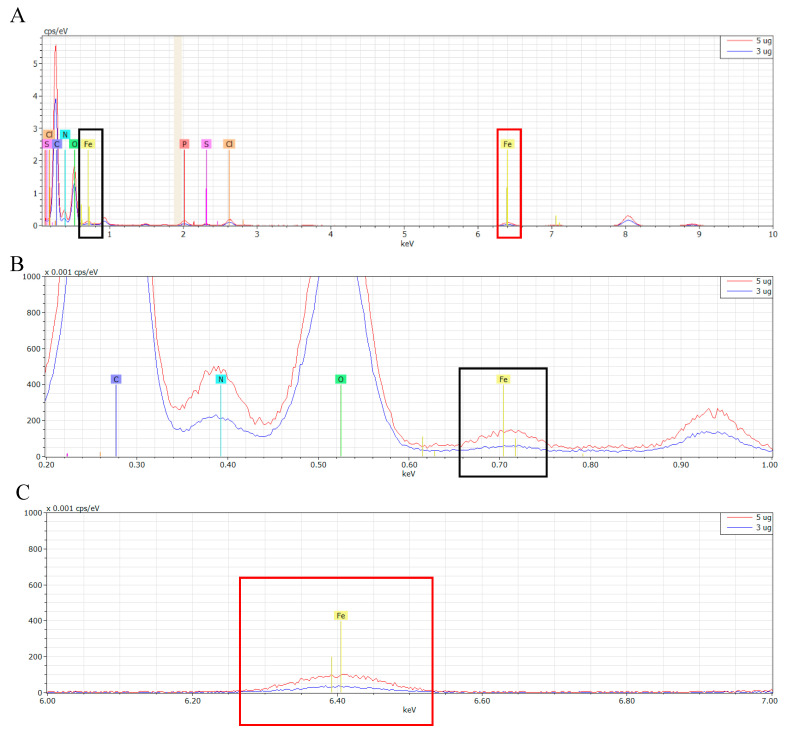
Overlapped spectra of the elemental mapping of nanoMIL-89. PAECs were treated with 3µg/mL (blue line) and 5µg/mL (red line) of nanoMIL-89, respectively. (**A**) showing the full spectra of elements signal (cps/eV) with respect to energy signal (keV), zoomed in (**B**) to show the L⍺ Fe signal (≈0.705 keV) and in (**C**) to show the K⍺ Fe signal (≈6.398 keV) for both samples. The intracellular iron signal was measured using Bruker EDS installed in a Talos TEM system.

The cellular uptake of NPs was further assessed by STEM (200 kV). Results showed that nanoMIL-89 was taken up by the cells and then packaged internally in the cell endosomes and lysosomes (Figure 9B,E), as reported previously by Carenza et al. [28] and as illustrated in Scheme 1. Indeed, iron oxide NPs were shown to form large aggregates (500 nm–2 µm) when placed in tissue culture media, as seen in TEM images of bare nanoMIL-89 (Figure S4). Moreover, EDS analysis was performed to corroborate the formation of nanoMIL-89 aggregates by carrying out an elemental mapping, as indicated in Figure 9C,F, and Supplemental Data (Figure S5). Figure 10 shows the iron (Fe) content in PAECs treated with 3 µg/mL and 5 µg/mL of nanoMIL-89. The Fe signal in L-alpha shell (L⍺) and K-alpha shell (K⍺) was 191.85 and 119.66 cps/eV (for 3 µg/mL), and 202.84 and 125.76 cps/eV (for 5 µg/mL), respectively (Figure 10B,C).

## 4. Discussion

Cardiovascular diseases are the leading cause of death in the world, killing over 18 million individuals per year [29]. The world health organization estimates an increase in the number of deaths from CVD to 24 million by 2030. The etiology of CVDs is complex and involves the interaction of genetic, environmental, and lifestyle factors. Despite their diverse origins, the shared features are endothelial dysfunction, smooth muscle cell proliferation and vascular remodeling. Current therapies are based on improving the endothelial-dependent vasodilator pathways, and inhibiting the vasoconstrictor pathways. However, these drugs are not capable of reversing the disease in humans, and systemic side effects hinder their long-term use. This implies that the problem might not reside solely in the drug efficacy, but rather in the pharmacokinetics and dosage. Taking into account the complexity of the human body, a more targeted drug delivery approach needs to be implemented for hitting the affected site with the optimum drug concentration. Trapping drugs in delivering vehicles (i.e., nanoparticles) can serve this purpose and also avoid the side effects seen in other tissues and organs. Moreover, if the right nanoparticle is used, the drug’s efficacy could be further increased, as seen in other conditions like cancer [30].

Of the different MOF nanoparticles used, nanoMIL-89 has shown promising therapeutic features. It provides more room for innovation, and because of being traceable, it can be used to monitor disease progression. When introducing a new nanoparticle for drug delivery, it is first essential to determine its interaction and cellular responses within the cells relevant to the studied disease. Endothelial and smooth muscle cells are the main culprits involved in the development of CVDs, thus representing valuable biomarkers and therapeutic targets. Therefore, in this study, we have investigated the effect of nanoMIL-89 on the viability and cytotoxicity of PAECs and PASMCs, and then we studied the cellular uptake and cytoplasmic localization within the cells. Our results showed that nanoMIL-89 was relatively non-toxic to both PASMCs and PAECs at moderate to low concentrations, respectively. This is consistent with our previously published results demonstrating the harmlessness of nanoMIL-89 on a range of human and murine cells [23].

Furthermore, it is well known that under inflammatory conditions, endothelial cells release high levels of the chemokine CXCL8. Here, we showed that CXCL8 release from PAECs was significantly reduced when treated with nanoMIL-89, suggesting that these NPs might have anti-inflammatory effects. These findings are in accordance with the previously published results by Saeidienik et al., who showed that iron NPs exert anti-inflammatory and neuroprotective effects in rats [31]. The fact that nanoMIL-89 exerts anti-inflammatory effects on PAECs is auspicious, as inflammation is a key component in the development of many CVDs. However, this mechanism requires further investigation. Having an NP subtype that can be taken up by endothelial and smooth muscle cells will be highly beneficial for future therapies of CVDs. Therefore, in this study, we monitored the internalization and localization of nanoMIL-89 in PAECs and PASMCs under normal and inflammatory conditions, and demonstrated their stability within the cells, as they were detectable in the cell cytoplasm seven days following their internalization.

Interestingly, we found that once nanoMIL-89 is taken up by the cells, it accumulates in the endosomes and lysosomes of the cells, as shown by STEM. This was confirmed by showing that the sizes of the accumulated NPs within the cells were larger (5-fold) than the original size of NPs upon preparation. This is due to the fact that once placed in endothelial cell culture media, the iron NPs are affected by ionic interactions and increase in size by forming large aggregates. Our findings are in accordance with the study reported by Carenza et al., which investigated the use of an iron NP (super magnetic iron oxide NPs; SPIONs) for the early detection of endothelial progenitor cells [28]. The cellular uptake of nanoMIL-89 by these cells is considered to be a great advantage for future drug delivery approaches, as it rules out the possibility of nanoparticles being eliminated by the reticuloendothelial system [32]. Therefore, the nanoMIL-89 cellular uptake will prolong the bioavailability of the encapsulated drug and, when aided with targeting mechanisms, will ensure its delivery to specific target sites.

The specificity of nanoMIL-89 can be further enhanced by several approaches. A primary drug-nanoparticle targeting strategy is coating the nanoparticles with ligands, which will result in a specific cellular uptake [33]. A new technique, known as mechanotargeting, works on improving the specificity of NPs’ cellular uptake. Mechanotargeting strategies deal with modifying cell surface mechanics to enhance biased cellular uptake. However, this targeting strategy is still under investigation [34]. Following its cellular uptake, nanoMIL-89 was also shown to transfer to daughter cells during cell division. This confirms that the drug would not only be present in mother cells but also in the multiplied cells, which will enable the tracking of studied cells as well as the prolonging of the effect of the half-life of the loaded drug, as well as minimizing the elimination and clearance by the immune system.

In summary, our findings underscored the suitability of nano-MIL-89 as a next-generation drug carrier in CVDs. Ultimately, such a development will set the foundation for the establishment of the nanoMIL-89-based drug delivery platform in the management of debilitating forms of CVDs such as the PAH.

## 5. Conclusions

Nanotechnology is considered an emerging branch in science that has various applications in different fields, including environmental, engineering, and biomedical applications. Nanomedicine has introduced new advancements in drug delivery, using particles in the 1–150 nm size range. It is believed that the development of nanoparticle-based drug formulations would yield the opportunities to address and treat challenging incurable diseases. The results of this study have underlined the role of nanoMIL-89 as a promising prototype to be used in achieving targeted drug therapy. However, for wider application of nanoparticles in clinical practice, more in vivo studies and pre-clinical trials are needed to understand the toxicity, long-term biological responses, and pharmacodynamics of nanoparticles. Furthermore, the selection of a validated and predictive animal model for nanoparticle-based drug delivery and therapy is pivotal to bridge the translational gap to the clinic. Finally, the innovation of nanotechnology and the outcomes of pre-clinical trials may represent a proof-of-concept of nanoMIL-89 to be used not only for CVDs treatment, but also for other incurable diseases such as cancer, diabetes, and Alzheimer’s disease.

## Supplementary Materials

The following are available online at https://www.mdpi.com/2079-4991/10/6/1028/s1, Figure S1: Orthogonal sections of Z-stack images. (**a**) Frontal (xy), (**b**) Transverse (xz)and (**c**) Sagittal (yz). Figure was produced by Zen blue software; Figure S2. nanoMIL-89 cellular uptake by PAECs and PASMCs treated with 1µg LPS. Cells were treated with different nanoMIL-89 concentrations (0, 1, 3, 10, 30 and 100 µg/mL) in a 1 µg/mL LPS-treated media. Images were then taken using light microscope at a magnification of 20× at three timepoints that range from day 1 to day 7; Figure S3. Blind scoring of nanoMIL-89. Cells were treated with different concentrations of nanoMIL-89 (1, 3, 10, 30, 100 µg/mL) in inflammatory conditions (1 µg/mL LPS) and imaged with inverted light microscope. Cellular uptake of nanoMIL-89 by (**A**) PAECs and (**B**) PASMCs was determined by blinded visual scoring (n = 6 independent assessments) according to a relative scale from 0 to 5; Figure S4. TEM images of nanoMIL-89 dissolved in dH2O. (**A**) and (**B**) TEM images of nanoMIL−89 aggregations (magnification 4000 × and 20,000×, respectively) forming spindle shape aggregates with 500 nm–1 µm diameter; indicated by the red arrows. These aggregates result from the crosslinking of single nanoparticles (80–200 nm) presented in (**C**) and (**D**) (magnification 38,000× and 295,000×, respectively); Figure S5. Energy dispersive spectroscopy elemental mapping showing iron, oxygen and carbon distribution of nanoMIL−89 in PAECs and PASMCs, showing the distribution of nanoMIL−89 particles inside the cells at a concentration of 5 µg/mL.
