# Supplementary material for: Internalization of Metal–Organic Framework Nanoparticles in Human Vascular Cells: Implications for Cardiovascular Disease Therapy
Source: Nanomaterials (Basel). 2020 May 27;10(6):1028. doi: 10.3390/nano10061028 (PMC7353612; doi:10.3390/nano10061028)
Supplement: Supplementary file 1 [file nanomaterials-10-01028-s001.pdf]

[Back Matter]

**Supplementary materials:**

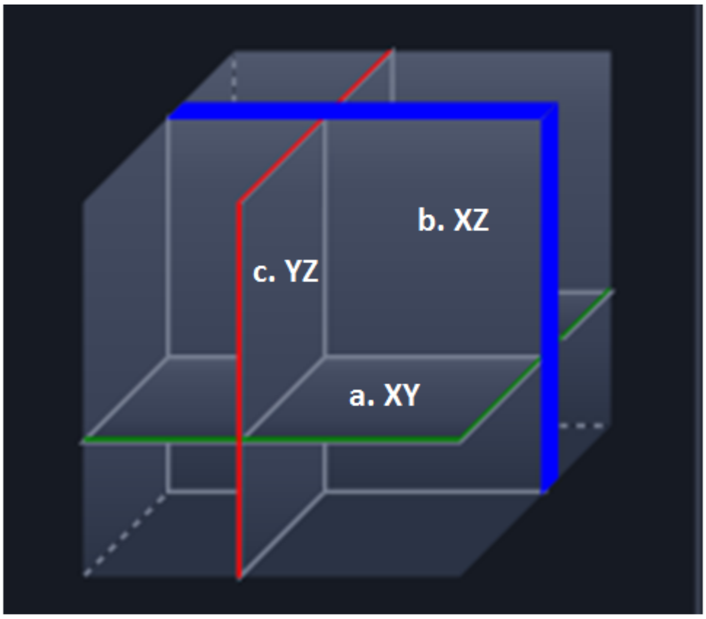


***Figure S1*** *Orthogonal sections of z-stack images. a) Frontal (xy), b) Transverse (xz)and c) Sagittal (yz). Figure was produced by Zen blue software.*

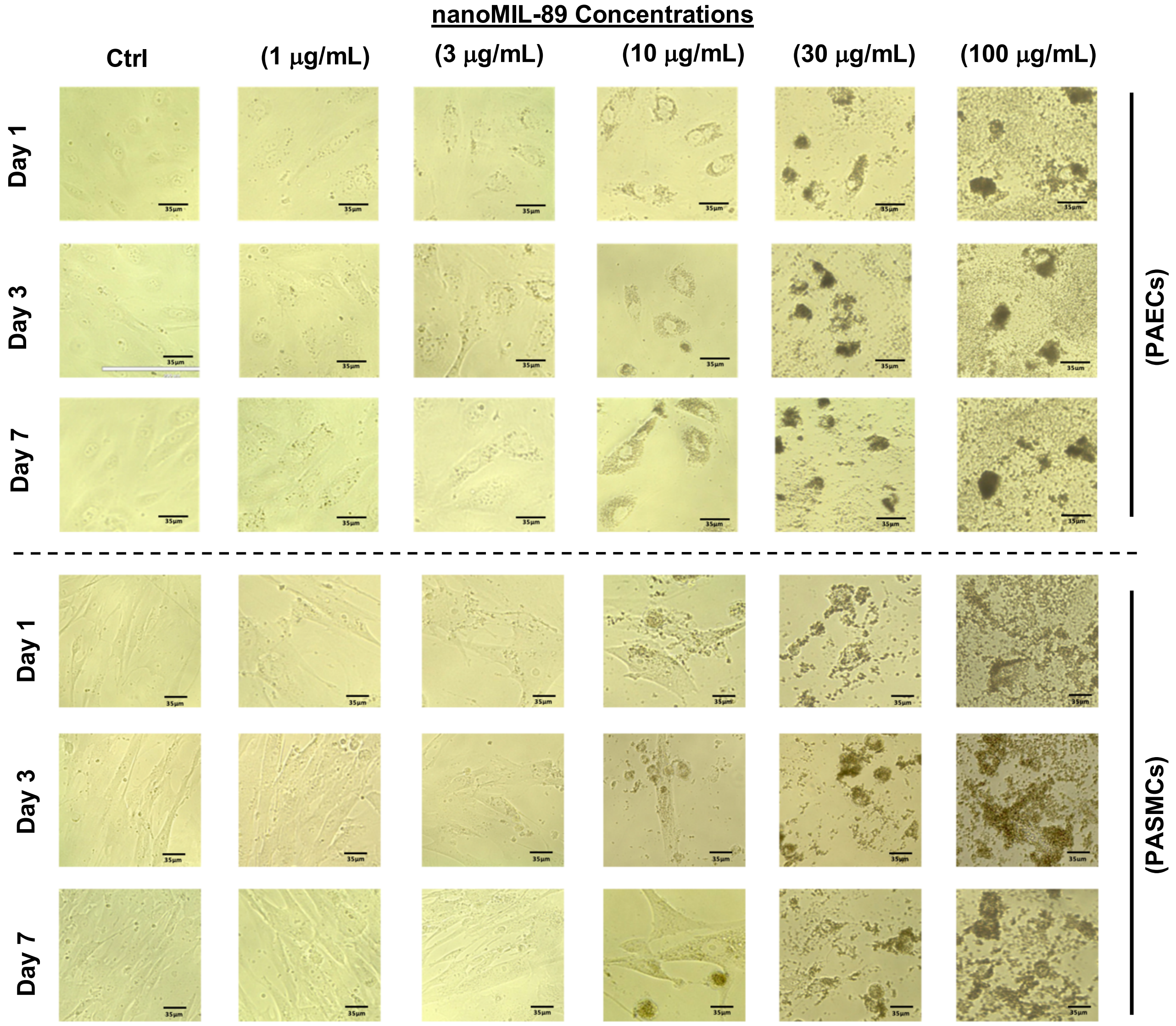


**Figure S2: nanoMIL-89 cellular uptake by PAECs and PASMCs treated with 1µg LPS.** Cells were treated with different nanoMIL-89 concentrations (0, 1, 3, 10, 30 and 100 µg/mL) in a 1 µg/mL LPS treated media. Images were then taken using light microscope at a maginification of 20x at three timepoints that ranges from day 1 to day 7.

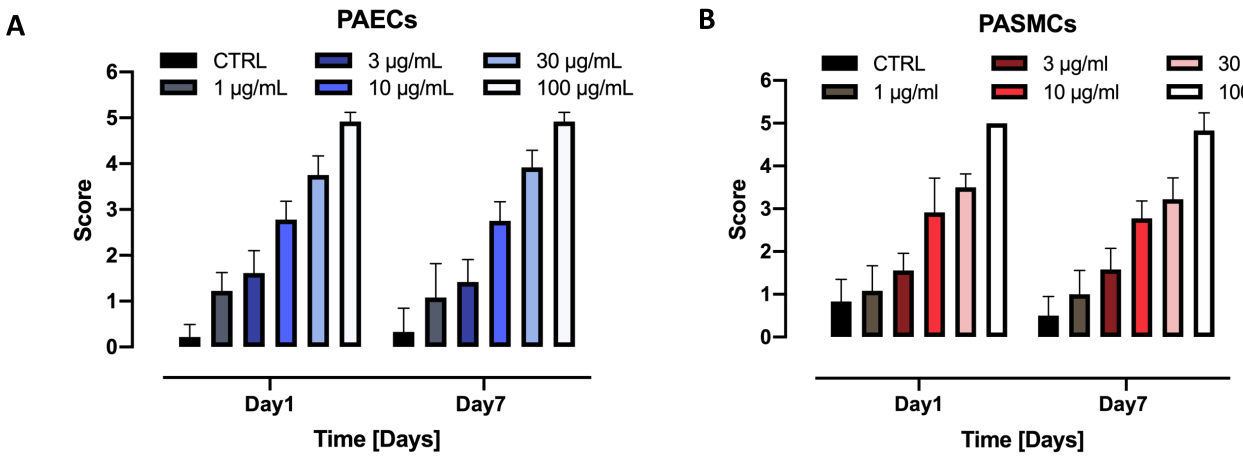


**Figure S3: Blind scoring of nanoMIL-89.** Cells were treated with different concentrations of nanoMIL-89 (1,3,10,30,100 µg/mL) in inflammatory conditions (1 µg/mL LPS) and imaged with inverted light miscroscope. Cellular uptake of nanoMIL-89 by (A) PAECs and (B) PASMCs was determined by blinded visual scoring (n = 6 independent assessments) according to a relative scale from 0 to 5.

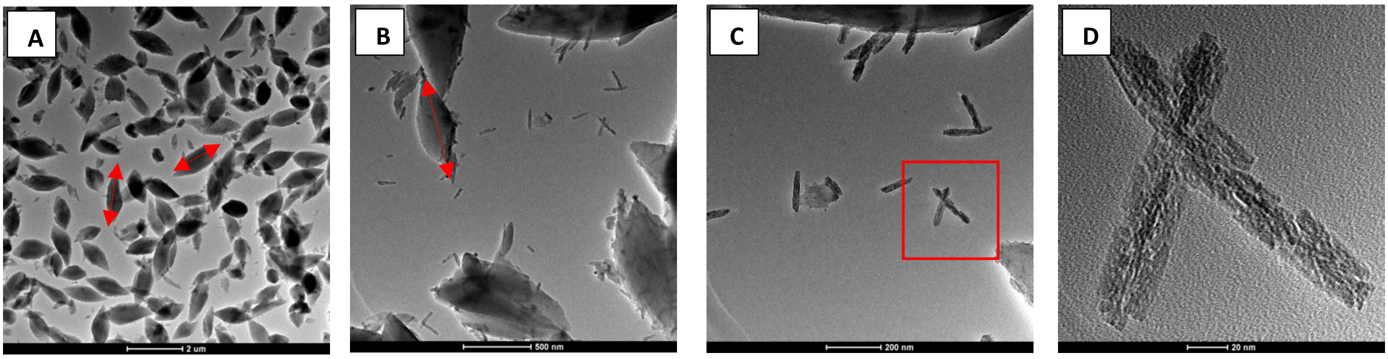


**Figure S4: TEM images of nanoMIL-89 dissolved in $dH_2O$.** (A and B) TEM images of nanoMIL-89 aggregations (magnification 4,000 x and 20,000x, respectively) forming spindle shape aggregates with 500 nm – 1 µm diameter; indicated by the red arrows. These aggregates result from the crosslinking of single nanoparticles (80 – 200 nm) presented in C and D (magnification 38,000x and 295,000x ,

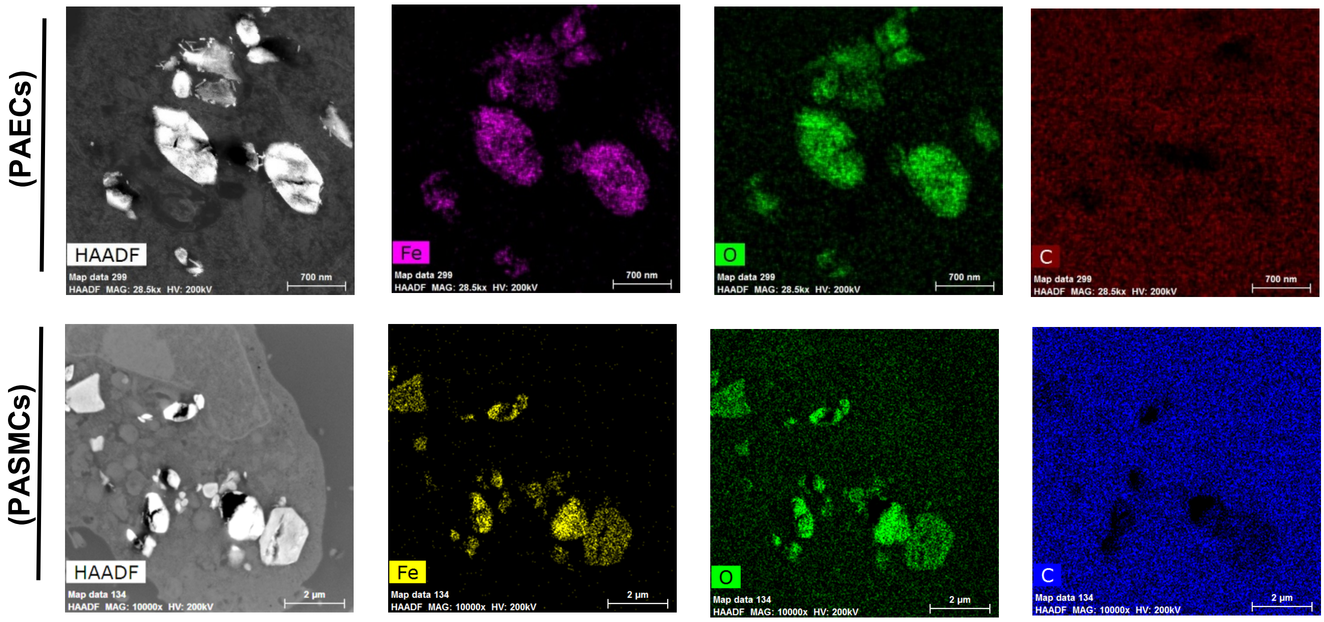


**Figure S5:** Energy dispersive spectroscopy elemental mapping showing iron, oxygen and carbon distribution of nanoMIL-89 in PAECs and PASMCs showing the distribution of nanoMIL-89 particles inside the cells at a concentration of 5 µg/mL.
